# Hepatoprotective effect of mulberry (*Morus nigra*) leaves extract against methotrexate induced hepatotoxicity in male albino rat

**DOI:** 10.1186/s12906-015-0744-y

**Published:** 2015-07-25

**Authors:** Hend M. Tag

**Affiliations:** Zoology Department, Faculty of Sciences, Suez Canal University, Ismaillia, 41522 Egypt

**Keywords:** Mulberry leaf extract, Methotrexate, Hepatotoxicity, HepG2 cells

## Abstract

**Background:**

Drug-induced liver injury is a major health problem that challenges not only health care professionals but also the pharmaceutical industry and drug regulatory agencies. The possible hepatoprotective effect of the administration of mulberry ethanolic extract (MUL) leaves against hepatotoxic effect of the anti-rheumatic drug, methotrexate (MTX) was evaluated in this study both vivo (using animal models) and in vitro (human hepatoma HepG2 cells).

**Methods:**

In the in-vivo study, 20 male albino rats were equally assigned into four groups; control group received distilled water orally; MUL treated-group received 500 mg/kg/day of MUL extract; MTX treated-group was injected with a single dose of 20 mg/kg MTX intraperitoneally on the 4th day; MUL-MTX treated-group received the previously mentioned doses of MUL and MTX (both control and MUL treated groups were administered a single dose of a physiological saline i.p.). At the end of the experimental period (14 days) activities of alanine transaminase (ALT), aspartate transaminase (AST), alkaline phosphatase (ALP) and lactate dehydrogenase (LDH) as well as total serum protein (TP) and albumin (ALB) levels were evaluated to assess liver function.

**Results:**

A marked reduction in the viability of HepG2 cells was observed after 48 h with IC_50_ equal to 14.5 μg/mL of MUL administration. Treating the animals with MUL in combination with MTX mitigated liver injury, causing a significant reduction in activities of AST, ALT, ALP and LDH as compared to the MTX-group. The liver architecture revealed more or less normal appearance with the combined treatment when compared with MTX treatment alone.

**Conclusions:**

This study recommends that the co-administration of MUL with MTX that may have therapeutic benefits against MTX-hepato-cytotoxicity.

## Background

Drug-induced liver injury is a major health problem that challenges not only health care professionals but also the pharmaceutical industry and drug regulatory agencies [1]. The drug-induced injury could be induced through different ways including direct toxic effect; immunological reaction or through active metabolite that is formed by the drug [2]. Methotrexate (MTX) drug, a structural analogue of folic acid, is widely used as anti-an rheumatic, cytotoxic chemotherapeutic agent for malignancies as well as in the treatment of various inflammatory diseases [3, 4]. MTX is actively accumulated in the liver where it is metabolized and stored in polyglutamated form, thus decreases folate levels by the inhibition of dihydrofolate reductase [5]. The widespread use of MTX and it is long-term therapy has attracted physicians’ attention to the possible adverse reactions of MTX [6].

Traditional plant medicines or herbal formulations might offer a natural key to hepatoprotective effect against xenobiotic/drug [7]. *Morus nigra* (Moraceae) is widely distributed in Asia, Africa, Europe, and America, and it is commonly known as black mulberry. It has been reported that *Morus nigra* is antioxidant and has protective action against oxidative damage to membranes and biomolecules [8, 9]. Important phyto-chemical constituents e.g. flavonoids, alkaloids and phenols have been reported in this plant [8, 10]. The flavonoids compound has shown to have hepatoprotective activity as reported previously by Adedosu et al. [11]. In addition, Naderi et al. [12] and Mallhi et al. [13] investigated the hepatoprotective effects of *Morus nigra* in both human and animals.

Therefore, the aim of the present work is to investigate the hepatoprotective effects of black mulberry leaf extract against the possible MTX-induced liver injury in human hepatoma HepG2 cells as well as in male albino rats.

## Methods

### Chemicals and plant materials

Methotrexate was purchased from Orion Corporation, Espoo, Finland. Other chemicals and reagents were of high analytical grade and were bought from standard commercial suppliers. The leaves of mulberry plant were collected from Ismaillia government, Egypt. The plant materials were identified from a taxonomist, Department of Botany, Faculty of Science, Suez Canal University, Ismailia, Egypt.

### Preparation of the plant extracts

Plant material was collected from mulberry trees which cultivated in Faculty of Science, Suez Canal University, Ismailia, Egypt, in March 2014 and were identified and authenticated by Botany Department, Faculty of Science, Suez Canal University on the basis of taxonomic characters and by direct comparison with the herbarium specimens with a voucher number (HERBFAS#5) available at the herbarium of Botany department. The plant extract was prepared as described before in [14, 15] with minor modifications. Briefly, the leaves of *Morus nigra* were washed, air-dried and powdered. The dried powder was extracted with 50 % hydro-ethanol solution for 48 h. The marc was further extracted by 50 % hydro-ethanol for 48 h to obtain the extract. The extract was then filtered and evaporated to dryness under reduced pressure on a rotary evaporator. The yield of ethanolic extract of *Morus nigra* leaves was found to be 10.2 % *w*/*w*.

### In vitro study: cytotoxicity evaluation using viability assay

HepG2 cell line (human cell line of a well differentiated hepatocellular carcinoma isolate from a liver biopsy of a male Caucasian aged 15 years) was obtained from the American Type Culture Collection (ATCC). Cells were cultured in Dulbecco’s Modified Eagle Medium (DMEM) with Earle’s salts supplemented with 10 % fetal bovine serum (Bio-Whittaker, Lonza, Belgium), 2 m M L-glutamine, 50 IU/mL streptomycin and maintained at 37 °C in humidified 5 % CO_2_.

Doxorubicin hydrochloride, an anticancer drug (VHB, Medi-sciences, Ltd, India) was used in the present study as a positive control. The drug was used within 15 days of purchase and was stored at the prescribed temperature. The dilutions were made in phosphate buffer saline (PBS).

Cell viability was assessed after 48 h of incubation with mulberry leaf extract or doxorubicin hydrochloride (50.0, 25.0, 12.50, 6.25, 3.12, 1.56 μg/mL) by crystal violet staining which dyes cellular nuclei. The staining was performed according to Henriksson et al. [16] method with slight modifications. Briefly, the cells were harvested, suspended in the growth medium mentioned above and seeded on 96-well plates (Falcon, NJ, USA) of 1 × 10^4^ cells per well. After 24 h, the cells were incubated at 37 °C in a humidified incubator with 5 % CO_2_ for 48 h with mulberry leaf extract or doxorubicin hydrochloride. The medium was then removed and the cells were fixed in situ with 4 % formaldehyde solution in PBS for 30 min at room temperature. The cells were washed twice with PBS (pH 7.4) and stained with 0.5 % crystal violet dissolved in 25 % aqueous solution of methanol for 5 min at room temperature. Unbound dye was washed out with deionized water and the cells allowed to air dry. The dye was solubilized in 33 % aqueous solution of acetic acid while shaking for 30 min at room temperature. Optical density (OD) was measured with a microplate reader (TECAN, Inc.), at wavelength of 490 nm.

### In vivo study

#### Animals and experimental design

Twenty male albino rats weighing 180–200 g obtained from the animal house of National Research Center, Egypt were used. Rats were housed in an air-conditioned room with 12-h light and dark cycles at 22 ± 2 °C. They were fed standard rodent diet with tap water *ad libitum*. Animals were divided into 4 groups, 5 rats each. The experiment was performed in accordance with the internationally accepted standard ethical guidelines for laboratory animal use and care as described in the European Community guidelines [17].

The first group ‘*control*-*group*’ was untreated with neither MTX nor MUL. The second group ‘*MUL*-*group*’ received mulberry leaf extract intragastrically at a dose of 500 mg/kg daily for 14 days [13]. The third group was injected with a single dose of MTX (20 mg/kg, I.P.) [18] at the fourth day of the experiment, ‘*MTX*-*group*’. The fourth group ‘*MUL*+*MTX*-*group*’ was administrated with MUL and MTX at the same does, described in second and third groups, respectively. Both control and MUL treated groups were administered a single dose of a physiological saline (IP). All animal treatments were conducted daily for 14 days.

#### Sample collection

At the end of the experiment, rats in all groups were anaesthetized with ether and blood samples were withdrawn from retro-orbital venous plexus by a capillary tube. Serum was separated followed by centrifugation at 4000 rpm for 10 min and stored at −20 °C until the biochemical analyses were performed. The animals were then euthanized by cervical dislocation before sacrificing. Livers were dissected out, blotted, dried and weighed. For each group livers were fixed in 10 % formalin saline for routine histopathological examination.

#### Assessment of hepatic function markers

The activities of alanine aminotransaminase (ALT) and aspartate aminotransaminase (AST) were determined in serum using commercial kits (Human Company, Germany, Cat. No. EC 2.6.1.2), based on kinetic method of Schumann et al. [19]. Lactate dehydrogenase (LDH) activity was evaluated using the method of Young [20] (Biosystems Company, Spain, Cat. No. M11580i-10). Alkaline phosphatase (ALP) activity was determined using commercial kit (Pointe Scientific, Inc. Brussels, BELGIUM, Cat. No. P803-A7516-01), based on the recommended method of Tietz [21]. Total protein (TP) and albumin (ALB) levels were measured using commercial kits (Human Company, Germany, Cat. No. INF. 157001. GB and INF.156001.D, respectively).

#### Morphometric and histopathological study

##### Determination of hepatosomatic index

The gross liver weight of each rat was determined using an electronic balance immediately after the freshly harvested liver was blotted dry. A hepatosomatic index (HSI) was computed by expressing the liver weight as a percentage of the rat’s body weight at the end of the experiment [22].

##### Histopathological evaluation and image analysis

Liver specimens were quickly removed, immersed in 10 % formalin, dehydrated and embedded in paraffin, sectioned at 4 μm, stained with hematoxylin and eosin (H&E) and evaluated by light microscopy. The histopathological scoring analysis was performed according to Lobenhofer et al. [23], the assessment was expressed as the sum of the individual score grades from 1 (minimal), 2 (mild), 3 (moderate), to 4 (marked) for each of the following parameters from liver sections: hepatocyte necrosis, fibrosis (collagen deposition), cellular infiltration, hepatocyte apoptosis and hepatocyte fatty change.

##### Identifying and quantifying liver fibrosis

The liver tissue samples were fixed in 10 % neutral buffered formalin, embedded in paraffin, sectioned at 4 μm thicknesses and stained with Goldner’s Masson Trichrome for detection of collagen fibers.

Ten colored histological images resulting from Goldner’s Masson Trichrome staining were captured by a digital camera (Canon Power Shot A640) and digitalized at 1024 × 768 pixel, 24 bit/pixel resolution, from 10 non-overlapping random fields per histological section in randomly chosen three animals from each group, at ×200 magnification. The digital images were processed using the image J (version 1.32j, the national institution of health, USA) analysis software. The image J automatically identifies and isolates fibrotic areas according to their staining (green) and then measures the area occupied by fibrosis with respect to the total liver area examined [24]. The percentage of area occupied by fibrosis (PF) is then determined, according to the following equation, PF = (labeled fibrosis area/total image area) × 100 [25].

### Statistical analysis

All values were expressed as mean ± standard error (X ± SE). Comparisons of evaluated parameters between the experimental groups were made using the one-way analysis of variance (ANOVA). Post-hoc comparisons were conducted by Duncan procedure. Statistical analyses were performed with the Statistical Package for the Social Sciences (SPSS), version 20.0 for windows (SPSS Inc., Chicago, IL, USA), with a significant difference of *p* < 0.05.

## Results

### Influence of MUL on viability of HepG2 cell line

**Fig. 1:**
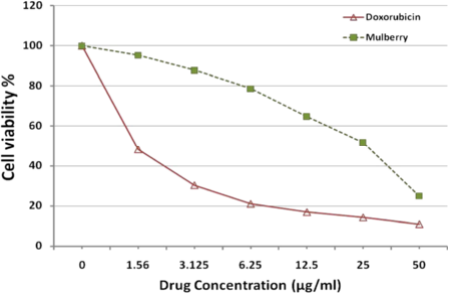
Effects of mulberry leaf extract on HEPG2 cell line using viability assay

The effects of ethanolic extract of Mulberry leaf against human HepG2 cells are shown in Fig. 1. Concentration above 12.5 μg/mL resulted in a marked reduction in cell viability. The percentage of growth inhibition was found to increase with increasing concentration of the test compound. The IC_50_ values of MUL and Doxorubicin hydrochloride found to be 14.5 and 1.2 μg/mL respectively.

### Effect of MUL extract on MTX-induced hepatotoxicity

The biochemical parameters of AST, ALT, ALP, LDH activities as well as the levels of TP and ALB were measured to estimate the general liver function. As shown in Table 1, animals treated with MUL did not show noticeable changes compared with control group. Whereas, the administration of MTX induced hepatotoxicity indicated by the increase in ALT, AST, ALP and LDH activities compared with control group. Serum total protein and albumin levels displayed no significant difference as compared with control. On the other hand, rats treated with both MUL and MTX in combination had significant decrease in ALT, AST and LDH activities compared with MTX group (*p* < 0.05).

**Table 1:** Effects of ethanol extract of *mulberry* on various biochemical parameters in rats with chemically induced liver toxicity

Groups	ALT activity (U/l)	AST activity (U/l)	ALP activity (U/l)	LDH activity U/l	Total protein g/dl	Albumin g/dl	Albumin to Globulin Ratio. (A/G) g/dl
Control	40.50 ± 3.28	93.25 ± 2.36	66.00 ± 7.12	470.50 ± 9.14	7.100 ± 0.163	4.13 ± 0.16	1.50 ± 0.04
MUL	19.50 ± 1.19	62.00 ± 5.79	36.25 ± 9.25	422.25 ± 12.25	7.00 ± 0.73	4.03 ± 0.06	1.43 ± 0.03
MTX-	71.00 ± 12.34^a^	135.75 ± 25.55^a^	110.25 ± 13.82^a^	1607.25 ± 121.57^a^	6.63 ± 0.10	3.83 ± 0.33	1.20 ± 0.08
MUL+ MTX	21.75 ± 3.25^b^	82.00 ± 6.00^b^	99.00 ± 7.55^b^	393.00 ± 87.18^b^	6.93 ± 0.048	3.70 ± 0.29	1.35 ± 0.23

Values are expressed as mean ± S.E. for five rats in each group

^a^Significantly different from control using one way ANOVA followed by Duncan

^b^Significantly different compared with MTX-treated group using one way ANOVA followed by Duncan

**Fig. 2:**
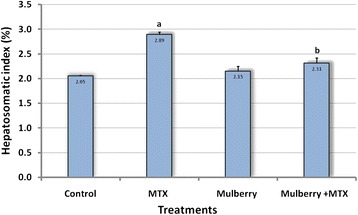
Effect of mulberry leaf extract on the hepatosomatic index (HSI) of MTX treated rats. Results are expressed as mean ± S.E. (*n* = 5), ^*a*^Significantly different from control using one way ANOVA followed by Duncan. ^*b*^Significantly different compared with MTX-treated group using one way ANOVA followed by Duncan. Hepatosomatic index: (Liver weight × 100)/body weight

Liver-body weight ratio (HSI) has been found to be significantly increased after MTX administration as compared with control group. However, there was no significant difference in HSI of MUL-MTX group when compared with control group, while there was apparent significant decrease in HSI values comparing MUL-MTX group with MTX-treated animals (Fig. 2).

**Fig. 3:**
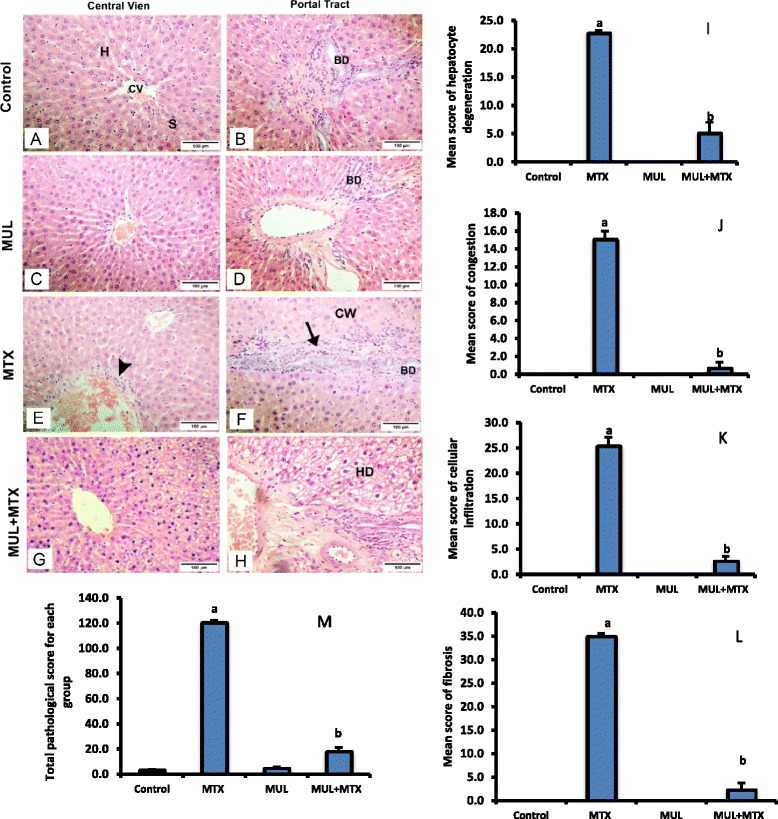
Hematoxylin and eosin-stained sections of rat liver: **a** and **b** Shows the general morphology of the classic liver (hepatic) lobule. It is roughly hexagonal in shape. At the *center* of the lobule is the central vein (CV). The hepatocytes (H) are organized into anastomosing cords or plates, one cell thick, separated by anastomosing hepatic sinusoids (S).; **c** and **d** Mulberry treated rat showed normal structure of heptic tissue. **e** and **f** Methotrexate (20 mg/kg) treated rat; **e** shows focal area of necrosis (*head arrow*) around the central vein where the cell boundaries were lost. **f** Shows heavy cell infiltration associated with fibrosis around the portal tract (*arrow*) and cloudy swelling of hepatocytes (CW). **g** and **h** Liver section from rats pre-treated with 500 mg/kg mulberry leaf extract and challenged with MTX showed preserved periventricular (cv) structure, absence of fibrosis, while noticing mild hydropic degeneration (HD) of hepatocytes. *Bar* = 100 μm. *Bars* represent mean ± SEM of histopathological scoring of the effect of mulberry leaf extracts on induced MTX-hepatic toxicity. ^*a*^ indicates significantly different values when comparing control animals, *p* < 0.05. ^*b*^ indicates significantly different values when comparing to MTX-group, *p* < 0.05

Results from histopathological studies provided supportive evidence for the biochemical analysis. The ‘*control group*’ (Fig. 3a–b) and ‘*MUL group*’ (Fig. 3c–d) showed a normal appearance of the liver cells, whereas the MTX-treated animal showed changes in hepatic architecture with centrilobular hepatic necrosis, cloudy swelling of hepatocytes and cell infiltration associated with fibrosis around the portal tract (Fig. 3e and f). Treatment with 500 mg/kg b.wt ethanol extract of MUL showed moderate enhancement in protecting liver cells from MTX-injury as shown in Fig. 3g and h.

The microscopic damage score for each group was determined, and illustrated in Fig. 3i–m. The hepatocyte degeneration for ‘*MTX group*’ (22.7 ± 0.33) and ‘*MTX*-*MUL group*’ (5.0 ± 1.15) was significantly increased compared with the ‘*control group*’ (Fig. 3i). The congestion in ‘*MTX group*’ (15.0 ± 1.00) was significantly increased, compared with the control group (0.00 ± 0.00), while there was no significant difference between MTX-MUL group and control group observed (Fig. 3j). The leukocyte infiltration in MTX group (25.33 ± 1.76) was significantly increased as compared with control group. However, there was no significant difference between MTX-MUL (2.67 ± 0.88) group and control group (0.00 ± 0.00) (Fig. 3k). The fibrosis in MTX group (35.0 ± 0.58) was significantly increased than control group, whereas there was no significant difference between MTX-MUL group (2.33 ± 1.45) and the control group (0.00 ± 0.00) (Fig. 3l). The total histopathology score for the liver was significantly increased in MTX-treated group (120.3 ± 1.76) compared with the control group (18.0 ± 3.21) (Fig. 3m), with no significant difference between MUL-group and control group.

**Fig. 4:**
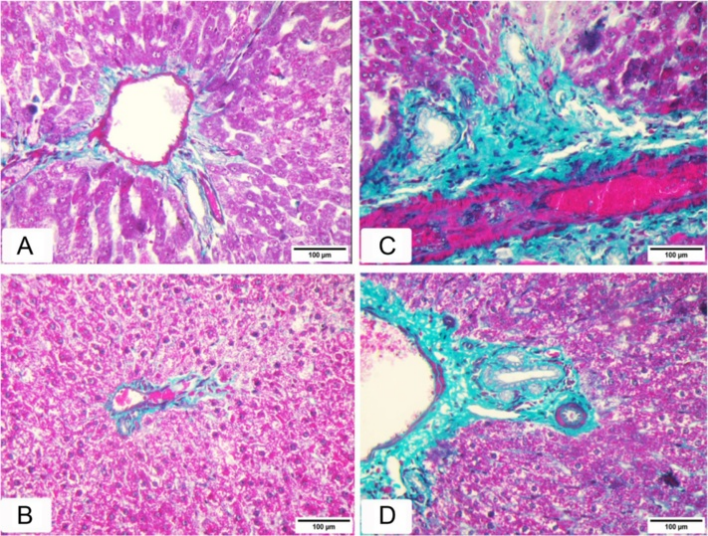
Masson’s Trichrome staining of collagen within the liver tissue. **a**, **b** Shows normal distribution of collagen fibers, *stained green*, around a portal tract in control and MUL-treated group, respectively. **c** Shows marked increase in collagen fibers around the blood vessels in the portal tract in MTX-treated group. **d** Shows normal distribution of collagen fibers, *stained green*, around a portal tract in MUL-MTX group, (Masson’s trichrome, ×200)

The results obtained from histological sections of livers with Masson’s Trichrome staining for control and MUL-treated rats were similar (Fig. 4a and b). The classic lobule was hexagonal in shape. The portal tract area was located at each corner of the lobule, and was composed of thin connective tissue septum containing few collagen fibers. Embedded in the portal tract there were a terminal portal venule, a terminal hepatic arteriole, an interlobular bile ductule and fine lymph vessels. The livers of the MTX treated animals showed an apparent increase in the amount of collagen fibers particularly around blood vessels in portal tract (Fig. 4c). Section of liver tissue from animals that were treated with combination of MTX and MUL showed less amount of collagen fibers (Fig. 4d) as compared with MTX-group. Quantification of fibrosis using semi-automated image analyses revealed a significant increase of fibrosis in liver of MTX-treated rats compared to control (19.50 ± 6.69 % vs1.33 ± 1.51 %, Table 2), and a significant decrease in MTX-MUL group compared to MTX-treated rats (2.64 ± 2.15 vs 19.50 ± 6.69, Table 2).

**Table 2:** Morphometric quantitative measurements of liver fibrosis in animal groups stained with Masson’s trichrome stain

Groups	Area %
Control	1.33 ± 1.51
Mulberry-Treated	1.22 ± 0.92
MTX-treated	19.50 ± 6.69^a^
Mulberry + MTX	2.64 ± 2.15^b^

Area %: represent the degree of hepatic fibrosis, the results are represented as mean value ± SE

^a^Significantly different from control using one way ANOVA followed by Duncan

^b^Significantly different compared with MTX-treated group using one way ANOVA followed by Duncan

## Discussion

Methotrexate (MTX), a folic acid antagonist, is widely used as a cytotoxic chemotherapeutic agent for several malignancies and various inflammatory diseases, which may lead to liver hepatotoxicity due to the depletion of the folate [26]. The results of the present study indicate that MTX caused increased AST, ALT, ALP and LDH activities. These results are in agreement with Tunali-Akbay et al. [27] who demonstrated that rats treated with MTX exhibited a toxic effect on liver where it provoked a notable elevation in serum activities of ALT and AST. ALT is a cytosolic enzyme of the hepatocyte and its increased serum activity reflects a leakage in plasma membrane permeability, which in turn, associated with cell death. ALT is considered to be one of the best indicators of liver necrosis [28]. Many authors describe the mechanism through which MTX induced hepatotoxicity. Jahovic et al. [26] and Al-motabagani et al. [29] reported that MTX can bind to the enzyme hydrofolic reductase, which bans conversion of folic acid to folinic acid causing block in some amino and nucleic acids synthesis. This might lead to damage of organelles and plasma membranes of hepatic parenchymal cells interfering with their function and allowing leakage of enzymes. Moreover, Kose et al. [30] stated that MTX caused increasing in malondialdehyde (MDA) and myeloperoxidase (MPO) levels leading to lipid peroxidation which is considered to be an important cause of destruction and damage to cell membranes, which has been suggested to be a contributing factor to the development of methotrexate-mediated tissue damage.

The present biochemical results suggest that MTX injection did not affect levels of TP, ALB and A/G ratio. The results from MTX treated groups was similar to those from the MUL+MTX treated group and control group. This could be possibly explained as Doumas and Peters [31] reported that any decrease of serum TP and ALB levels might indicate insufficient hepatocyte biosynthesis. One of the major reasons for a decline in biosynthetic functions is the low protein feeding diet. However, hence animals in the present study were fed a diet with suitable protein content, may be the cause of weaker variation in serum TP and ALB levels.

The biochemical changes induced by MTX treatment were confirmed by morphometeric and histological studies. Hepatosomatic index (HSI), is fundamental to liver injury diagnoses [32]. The liver indices of the experimental rats were noticeably affected by MTX treatment and regular oral administration of mulberry leaves extract. The results from this study show significantly higher HSI in MTX-treated rats when compared to control. This increase in liver weight may be probably due to formation of reactive oxygen species (ROS) which lead to hepatic damage [33] and liver inflammation [34]. MTX leaded to histopathological changes including hydropic degeneration, cellular infiltration and collagen deposition. These results are confirmed with other previous studies. Soliman [35] demonstrated that rats received a single injection of MTX (20 mg/kg, i.p.), exhibited a toxic effect on liver with plenty of focal sinusoidal areas, portal track inflammation, proliferation of Kupffer cells, focal liver cell necrosis, fibrosis and fatty changes in hepatocytes. Regarding, the Masson’s Trichrome staining, there was a progressive increase of collagen deposition after MTX injection. The present observation is in agreement with those of Ros et al. [36] who found perisinusoidal fibrosis due to direct toxic effects of MTX. Also, Hytiroglou et al. [37] stated that the MTX is known to cause hepatic fibrosis in some patients, which might progress to cirrhosis. MTX is known to cause lipid peroxidation through generation of ROS which damage the mitochondrial and cytoplasmic membranes causing more severe oxidative damage in the tissues [27]. This liver cell injury usually initiate activation of blood-derived phagocytes that increases the concentration of fibrogenic cytokines and recruit a lot of fibroblasts and fibroblast-like cells for excess production of extracellular matrix [27, 38].

According to the present results, the in vitro level of MUL exhibited inhibition on HepG2 cell line proliferation with IC50 equal to 14.5 μg /ml. These results are in agreement with previous results on HepG2 cells, showing that mulberry leaf extracts can inhibit cell proliferation and depress the levels of alpha-fetoprotein (AFP) and ALP in HepG2 cells than controls in a time dependent-manner [39], as well as through G2/M phase arrest and induction of apoptosis [40].

With regard to in vivo study, the administration of ethanolic mulberry leaves extract did not show any adverse affect on liver weight. The administration of MUL enhanced the hepatosomatic index of MTX treated rats and brought this parameter back to values very similar to that observed in the control group. Also, MUL given during the MTX application provided significant protection from the hepatotoxicity of MTX. Treatment with MUL in combination with MTX apparently exhibited a significant decrease in AST, ALT, ALP and LDH activities. Recently, Mallhi et al. [13] reported the hepatoprotective role of MUL on paracetamol induced liver injury in mice. Also, protective effect of MUL on rat liver injury in diabetic rats was demonstrated by Nazari et al. [41]. These studies showed that black mulberry administration prevented ALT and AST increase and improved liver function in diabetic rats. Moreover, Agha et al. [42] concluded that MUL juice possess antioxidant properties which could prevent liver dysfunction induced by CCl4. Thus, the possible mechanism may be due to free radical scavenger and antioxidant activities of MUL constituents, especially flavonoids (i.e., quercetin, rutin, and isoquercitrin) [43, 44]. According to the present results, liver sections of the rats treated with MUL at a dose of 500 mg/kg followed by MTX intoxication apparently ameliorated the gross and histological alterations in hepatic tissue induced by MTX. A possible mechanism of *M. nigra* extract as hepatoprotective may be due to its antioxidant effect, which can impair the activation of MTX into the reactive form. Since flavonoids have hepatoprotective activities [45]. Mulberry leaves were shown to contain at least four flavonoids, including rutin [44, 46, 47]. Flavonoids have long been recognized to possess hepatoprotective and anticarcinogenic activities [45, 48]. Moreover, leaves of mulberry plants have been recently reported to have antioxidant effect as they contain alkaloid as well as flavonoids Radojković et al. [43]. In the present work MUL administration to the MTX group significantly decreased the hepatic collagen content and histopathological score. This results may be due to the presence of flavonoids which known to possess remarkable antioxidant properties capable of protecting normal cells from various stimuli-induced oxidative stress and cell death [49]. The possible mechanism that is responsible for the protection of MTX-induced liver damage by mulberry leaves extract may be due to its radical scavenger activity. By trapping lipid and peroxyle radicals, MUL could hinder their interaction with polyunsaturated fatty acids and would abolish the enhancement of lipid per-oxidative processes [50].

Although Methotrexate is often the most effective agent for treating severe rheumatoid arthritis, hepatotoxicity is a frequent complication of long-term methotrexate therapy, and can force discontinuation of this drug [51]. According to considerations cited above likewise imply that MUL may be administered in conjunction with methotrexate therapy to encounter its oxidative effect.

## Conclusions

In conclusion, the present study clearly demonstrates that MUL administration protected liver tissues, probably due to its antioxidant and cytoprotective characteristics. In addition, the administration of MUL could be used with chemotherapeutic agents in combination therapy with MTX-treatment.
